# Special Issue “Veterinary Anatomy and Veterinary Pathology: 2nd Edition”

**DOI:** 10.3390/life15091359

**Published:** 2025-08-27

**Authors:** Gheorghe Solcan, Carmen Solcan

**Affiliations:** 1 Internal Medicine Clinic, Faculty of Veterinary Medicine, Ion Ionescu de la Brad Iasi University of Life Sciences, 8 M. Sadoveanu Alley, 700489 Iasi, Romania; 2 Department of Preclinics, Faculty of Veterinary Medicine, Ion Ionescu de la Brad Iasi University of Life Sciences, 700490 Iasi, Romania; carmen.solcan@iuls.ro

Anatomy is a basic science for both human and veterinary medicine. Veterinary anatomy faces new challenges. There is a need to understand current morphological fundamentals of animals through modern imagistic methods, such as computed tomography (CT), magnetic resonance imaging (MRI), ultrasonography, etc., but there is also a need to study new, exotic companions, or wild animals. Veterinary pathology is continuously progressing; in parallel with the progress of humankind, new strategies of diagnosis, prophylaxis, and therapy of animal diseases are being developed, to the benefit of both animals and human health and welfare.

This Special Issue, “Veterinary Anatomy and Veterinary Pathology: 2nd Edition”, aimed to publish original research works, reviews, and case reports within these topics, highlighting their importance in the search for new diagnostic, prophylactic, and therapeutic strategies for animal diseases. This reprint consists of 12 articles that present recent insights and advancements in the fields of animal anatomy and pathology, authored by 77 researchers from various countries (Romania, Poland, Italy, South Korea, and USA), addressing contemporary issues through interdisciplinary and multidisciplinary methods, from the anatomy of a monkey to modern imagistic methods (computed tomography, endoscopy, ultrasonography, and electrophysiology) used in the diagnostic and treatment of various animal pathologies, or the biocompatibility of some new biomaterials on animal experimental models.

Mortonos et al. [1] provide a comprehensive overview of the anatomical features of the humerus in the African green monkey (*Chlorocebus sabaeus*), along with comparative and differential aspects of monkey osteology. The micromorphological findings offer valuable insights into this relatively under-researched primate, highlighting similarities with other primates regarding histological structures and detailing specific histomorphometric elements related to secondary periosteal bone formations (osteons).

Beginning from anatomy of healthy dog (*Canis familiaris*), Choi [2] succeeded to establish some correlations between hyperlipidemia-related diseases and thorax/thigh circumference ratio along with body condition score in dogs. According to author findings, triglycerides (TG) had a strong positive link with thigh circumference and a negative correlation with thorax/thigh value. While the thorax/thigh value was negatively correlated with the medial patella luxation (MPL) grade, total cholesterol (TC) was strongly positively correlated with thigh circumference. The Apolipoprotein E (ApoE) gene might be essential for thigh fat accumulation. Despite that, the thorax/thigh circumference ratio failed to be a new indicator comparable to the waist/hip circumference ratio in humans.

Computed Tomography (CT) is a modern imagistic method for the diagnosis of many internal diseases (especially those of the nervous system) in humans and companion animals, but it is cost-limitative for farm animals. Using CT scans to confirm the presence of cysts of *Coenurus cerebralis* in the brain of sheep with neurological symptoms [3], Olar et al. [4] have shown that plasma kynurenic acid levels, as determined by fluorescence spectroscopy [5], may be used as diagnostic indicators for sheep with chronic coenurosis, allowing practical application in early-stage diagnosis at the farm level.

MRI is an advanced imaging method, giving even more accurate results than CT in some pathologies, but the longer exposure period, under general anesthesia, leads to the risk of hypothermia [6]. Pavel et al. [7] have demonstrated that a hot water bottle placed beneath the abdomen and a blanket covering the entire body can prevent hypothermia in cats requiring a head MRI.

Endoscopy is another modern imagistic method for diagnostic and treatment of many diseases, both in human and in veterinary medicine, and endoscopic surgery became an increasingly popular, low invasive procedure. Laparoscopic nephrectomy is a procedure performed by Prządka et al. [8], and even if in previous studies, the ureter has been closed using mechanical clips or stitches [9], the authors have succeeded in demonstrating that it is feasible to close the renal vessels and ureter, in both cats and dogs, with vascular sealing tools, using advanced bipolar coagulation techniques. The ureter closure was proved by histopathological postoperatory exam.

Infrared thermography (IRT) is a method that evaluates peripheral blood flow and the heat emitted as a result, which is now utilized in human healthcare to noninvasively examine peripheral vascular issues like thrombosis, thromboembolisms, and various ischemic, inflammatory, or tumor-related conditions. The use of IRT for evaluating blood vessel issues in veterinary science is still in the early stages, with few research studies released [10]. Using IRT to assess the comparative thermal pattern of the thoracolumbar area in healthy dogs and dogs with acute intervertebral disc extrusion (IVDE), Zaha et al. [11] have identified notable variations in the thermal patterns observed in dogs suffering from IVDE, when compared to healthy dogs. They suggest using thermographic scans of the thoraco-lumbar region as a less invasive approach for diagnosing IVDE.

In the same line of non-invasive diagnostic techniques, Strichea et al. [12], using ultrasonography of the liver, identified multiple hyperechogenic strands diffusely dispersed throughout the parenchyma, giving it a “Swiss cheese” appearance, an aspect considered pathognomonic for a rare and severe condition in dogs—hepatocutaneous syndrome. So, authors recommend ultrasound assessment of the abdomen in dogs with dermatological symptoms compatible with hepatocutaneous syndrome.

Electroencephalography (EEG) offers a factual basis for diagnosing seizures and epilepsy; however, it is considered infrequently utilized in the field of veterinary neurology [13]. Ștefănescu et al. [14] have studied EEG in a pediatric dog with seizures consequent to portosystemic shunt (confirmed by ultrasonography and CT), concluding that the EEG recording displayed bilateral simultaneous bursts of three-phase waves, similar to non-convulsive status epilepticus. Authors recommend EEG as a complementary method of the diagnosis of portosystemic shunting, which might be useful in initiating the therapeutic protocol, before final confirmation using more laborious diagnostic methods.

Employment of animal models in translational studies for the development of bone tissue engineering and regenerative medicine is a relatively new research direction in which veterinary pathology is increasingly involved [15]. Investigating the osteogenic potential and toxicological tolerance of two original bioproducts, Ardelean et al. [16] proved the biocompatibility of the biomaterials for rats femoral bones. This study could open new research directions concerning the use of dental biomaterials in creating frameworks for the restoration of bone defects.

Corneal abrasion represents the predominant eye-related issue that arises during general anesthesia for nonocular surgeries. The pain experienced afterward can occasionally be more intense than the discomfort caused by the surgery itself [17], yet it frequently goes unrecognized in veterinary practice. Given that there is no established method for safeguarding the cornea during general anesthesia [17], Pavel et al. [18], investigating the lacrimal gland tear production in healthy sheep under general anesthesia, proved the protective effect of 1% hyaluronic acid ophthalmic gel, offering essential information to assist anesthesiologists in effectively overseeing the impact of general anesthesia related to safeguarding the cornea.

Toxicology is an important branch of veterinary pathology in continuous development. Ochratoxin (OTA) is very toxic and ranks among the most prevalent mycotoxins that contaminate animal feed globally [19], and the quantitative determination is laborious and expensive. Beia et al. [20] have developed a rapid and sensitive method for the quantification of OTA levels in maize, utilizing ultra-performance liquid chromatography coupled with fluorescence detection (UPLC-FLD). The method separates OTA from matrix interferences, thereby ensuring reliable identification at minimal levels, serving as a trustworthy method for monitoring mycotoxins to maintain feed security and safeguard both animal welfare and public health.

Copper is an essential trace element, playing an important role in many physiological processes: energy generation, immunity, body development, nervous system activity, and the formation of connective tissues, but it can become harmful in animals that are excessively exposed [21]. Domestic sheep are the species that show the highest sensitivity to copper toxicity, and to reduce the intoxication risk, inorganic salts were replaced in feed additives with organic ones. Even so, Pivariu et al. [21] firstly report a classical outbreak of chronic copper intoxication in sheep produced by feed with added copper bilysinate, but destinated for pigs, which are much more tolerant to copper. So, a mistaken change in destination of feed ingredients from one species to another is an important intoxication hazard.

In conclusion, this Special Issue has provided recent updates and important findings in veterinary anatomy and pathology, like new diagnostic and therapeutic strategies in various animal diseases, specifically advanced imaging techniques (CT in sheep coenurosis, MRI in cats, ultrasonography, and thermography), electroencephalography, modern surgery procedures (laparoscopic nephrectomy), biocompatibility of some new biomaterials, and prevention of some intoxications.
